# Environmental Exposures and Epigenetic Remodeling in Supraventricular Tachycardias: What Atrial Fibrillation Can and Cannot Tell Us

**DOI:** 10.3390/life16091506

**Published:** 2026-09-09

**Authors:** Ioannis Konstantinidis, Sophia Tsokkou, Antonios Keramas, Theodora Papamitsou

**Affiliations:** Laboratory of Histology-Embryology, Department of Medicine, Faculty of Health Sciences, Aristotle University of Thessaloniki, 54124 Thessaloniki, Greece; stsokkou@auth.gr (S.T.); antonios@auth.gr (A.K.)

**Keywords:** atrial fibrillation, epigenetics, DNA methylation, microRNA, air pollution, supraventricular tachycardia, gene–environment interaction, GWAS, histone modification, atrial remodeling

## Abstract

Supraventricular tachycardias (SVTs), including atrial fibrillation (AF), atrioventricular nodal re-entrant tachycardia (AVNRT), atrioventricular re-entrant tachycardia (AVRT), and focal atrial tachycardias, can possibly arise from the interaction of genetic predisposition and environmental exposures. While genome-wide association studies (GWASs) have identified 525 loci for atrial fibrillation and a small number of loci for AVNRT and accessory pathway-mediated tachycardia, the contribution of air pollution, lifestyle factors and psychosocial stress to epigenetic remodeling of atrial tissue remains insufficiently integrated into current mechanistic models of arrhythmogenesis. This review aims to synthesize evidence on how environmental exposures, including PM_2.5_, NO_2_, ozone, tobacco smoke, obesity, alcohol use, and physical inactivity, modulate epigenetic pathways relevant to SVT susceptibility, and to evaluate whether AF-derived epigenetic insights can be extrapolated to other SVTs. Thus, a narrative synthesis was conducted across studies examining environmental determinants, epigenetic mechanisms (DNA methylation, histone modifications, non-coding RNAs), and genetic susceptibility in SVTs. Literature from cardiac tissue studies, circulating epigenetic biomarker analyses, and mechanistic AF models was integrated to construct a unified gene–environment–epigenome framework. In atrial fibrillation, environmental exposures are consistently associated with epigenetic alterations affecting atrial electrophysiology, inflammation, oxidative stress and structural remodeling, and air pollutants and lifestyle factors modulate methylation signatures, histone-modifying enzymes and microRNA networks implicated in atrial conduction and re-entry. For AVNRT, AVRT and focal atrial tachycardia the evidential position is different. Large prospective cohort and case-crossover analyses now link air pollution to incident and acute supraventricular tachycardia, and genome-wide association studies have identified susceptibility loci for AVNRT and for accessory-pathway-mediated tachycardia, including one gene encoding a cardiac chromatin-remodeling protein; but no epigenomic profiling of nodal or accessory-pathway tissue has been reported, and no study has measured an environmental exposure, an atrial epigenetic mark and a non-AF SVT endpoint in the same participants. Twin data indicate that approximately 35% of SVT risk is attributable to genetic and 65% to unique environmental factors, which motivates a gene–environment–epigenome framework without validating its mechanistic detail outside AF. Mapping GWAS-identified loci onto environmentally responsive regulatory pathways identifies candidate convergence points between inherited risk and exposure-driven remodeling; for non-AF SVT, these are designated working hypotheses rather than established mechanisms. Air pollution and lifestyle factors are associated with supraventricular arrhythmia across the spectrum, and in atrial fibrillation there is direct evidence that they act, in part, through epigenetic reprogramming of atrial tissue. No epigenetic panel has been prospectively validated for the prediction of any supraventricular arrhythmia, and precision risk stratification therefore remains a research objective rather than a near-term clinical horizon. Integrating environmental exposure data with genetic and epigenomic profiling is nonetheless the most plausible route toward it. Future priorities include exposure-stratified, cell-resolved epigenomic profiling of atrial and nodal tissue, prospective validation of candidate circulating markers against incident arrhythmia, and replication in non-European populations and in both sexes.

## 1. Introduction

Supraventricular tachycardias (SVTs) represent a diverse spectrum of cardiac rhythm disorders that originate above the bundle of His, consisting of atrial fibrillation (AF), AVNRT, AVRT, and focal atrial tachycardias. Although often characterized clinically as benign nuisances, they impose a substantial global burden. The Global Burden of Disease Study 2021 recorded 4.48 million (95% uncertainty interval 3.61–5.70) incident cases of AF and atrial flutter worldwide in that year, together with 8.36 million disability-adjusted life-years and 0.34 million deaths, and identified elevated systolic blood pressure and elevated body mass index as the two leading attributable risk factors [1]. AF is a leading cause of ischemic stroke, heart failure and premature death [2,3]. That the principal attributable risks are themselves modifiable, and are patterned by environment and behavior, is the starting point for what follows.

The pathogenesis of SVTs is multifactorial. Over the past decade, GWASs have identified more than 100 genetic loci associated with AF, pointing to genes involved in cardiac ion channel function, transcription factor activity, and structural remodeling [4,5]. Concurrently, the global epidemiological transition, which is characterized by rapid urbanization, persistent air pollution, sedentary lifestyles, rising obesity rates, and increasing psychosocial stress, has been accompanied by a parallel rise in SVT burden, suggesting that non-Mendelian mechanisms are at work. The question is not whether environment matters, but how its signals are transduced into lasting molecular changes within atrial tissue.

Epigenetics, which can be defined as the study of heritable and environmentally responsive changes in gene expression that occur without alteration of the underlying DNA sequence, has emerged as a compelling framework for reconciling genetic susceptibility with environmental risk. DNA methylation, histone post-translational modifications, and non-coding RNAs constitute the principal layers of this regulatory architecture, and each has been shown to be modifiable by pollutant exposures, lifestyle factors, and metabolic stressors [6,7]. Yet the field of SVT epigenomics remains fragmented. The overwhelming majority of mechanistic insight derives from AF models, with virtually no epigenomic data on AVNRT, AVRT, or focal atrial tachycardias as distinct entities. This reflects deeper structural limitations in how electrophysiology research conceptualizes the etiology of non-AF supraventricular arrhythmias. Their substrates, such as dual AV nodal pathways, accessory conduction pathways, enhanced automaticity foci, are distinct from the diffuse atrial fibrosis and electrical remodeling that sustain AF, and the assumption that AF epigenomics maps cleanly onto these entities requires scrutiny.

This narrative review synthesizes evidence across the environmental determinants of SVTs, the epigenetic mechanisms through which these determinants operate, and the genetic landscape within which they act. We use AF as the primary mechanistic lens while explicitly interrogating the limits of that extrapolation. Our aim is to articulate a gene–environment–epigenome framework that can guide future tissue-specific epigenomic profiling studies and, ultimately, precision strategies for SVT risk stratification and prevention.

## 2. Materials and Methods

This is a narrative review, prepared and reported in accordance with the Scale for the Assessment of Narrative Review Articles (SANRA) [8]. We searched PubMed/MEDLINE and Web of Science from database inception to 31 July 2026, restricted to English-language publications. The core search combined three concept blocks with the Boolean operator AND. The arrhythmia block comprised (“atrial fibrillation” OR “supraventricular tachycardia” OR “atrioventricular nodal re-entrant tachycardia” OR AVNRT OR “atrioventricular re-entrant tachycardia” OR AVRT OR “accessory pathway” OR “Wolff-Parkinson-White” OR “atrial tachycardia” OR “atrial flutter”). The exposure block comprised (“air pollution” OR “particulate matter” OR “PM2.5” OR “nitrogen dioxide” OR ozone OR smoking OR “tobacco smoke” OR alcohol OR obesity OR “physical activity” OR sedentary OR “psychosocial stress” OR diet OR “environmental exposure”). The mechanism block comprised (epigenetic* OR “DNA methylation” OR “histone modification” OR “histone acetylation” OR chromatin OR microRNA OR “non-coding RNA” OR lncRNA OR “epigenetic clock” OR GWAS OR “genome-wide association”).

Records were eligible if they reported human or experimental data linking at least one environmental exposure, at least one epigenetic or genetic mechanism, and an atrial arrhythmic, electrophysiological or structural-remodeling endpoint. We excluded conference abstracts without extractable data, single case reports, non-English publications, and analyses superseded by a later report of the same cohort. Titles and abstracts were screened independently by two authors (I.K. and S.T.), and full texts were assessed against the same criteria; disagreements were resolved by discussion, with residual disagreement adjudicated by the senior author (T.P.). Reference lists of included articles and of major reviews were hand-searched, and forward citation tracking was performed for the principal epidemiological and epigenomic sources. Where several analyses of the same exposure–outcome pair were available, we preferred systematic reviews and meta-analyses for effect estimation and primary studies for mechanistic detail, and we prioritized human over animal data and larger over smaller samples.

Two properties of the retrieved evidence base determine the architecture of this review and are stated here rather than left to be inferred. First, atrial fibrillation accounts for the substantial majority of studies satisfying all three concept blocks simultaneously; AVNRT, AVRT and focal atrial tachycardia are represented almost exclusively by genetic-association and clinical-phenotyping studies, and we identified no dedicated epigenomic profiling of nodal or accessory-pathway tissue. Second, no study was identified that measured an environmental exposure, an atrial epigenetic mark and a non-AF SVT endpoint in the same participants. Atrial fibrillation is therefore used throughout as the primary disease model, and every inference extended to AVNRT, AVRT or focal atrial tachycardia is designated a working hypothesis rather than an established finding.

## 3. Environmental Determinants of SVT Susceptibility

### 3.1. Air Pollution

The epidemiological evidence linking ambient air pollution to AF and broader arrhythmia risk has strengthened considerably over the past decade, supported by multiple systematic reviews and meta-analyses [9]. An umbrella review by de Bont et al. synthesizing 56 systematic reviews and meta-analyses found consistent positive associations between short-term exposures to PM_2.5_, PM_10_, and NO_2_ and both arrhythmia hospitalization and AF incidence, although long-term exposure data for arrhythmia endpoints remained sparse relative to other cardiovascular outcomes (Table 1) [10].

Studies employing cardiac implantable electronic devices (CIEDs) to objectively capture AF episodes have been particularly informative. Liu et al., using a case-crossover design in Beijing, found that each 10 μg/m^3^ increase in PM_2.5_ was associated with a 3.8% (95% CI: 1.4–6.2%) increase in the odds of AF occurrence, an effect that was apparent within days of exposure [11]. Dahlquist et al., working in a lower-pollution Scandinavian environment where PM_2.5_ levels were well below current WHO guidelines, still observed a significant association between 48 and 96 h lag concentrations of PM_2.5_ and AF episodes, suggesting that there may be no safe threshold [12].

In a large Australian study of 82,575 AF emergency department presentations over seven years, Dawson et al. found that high levels of NO_2_ (≥16.5 ppb) and PM_2.5_ (≥57.7 μg/m^3^) were independently associated with increased AF presentations, with attributable fractions of 7.24% and 3.81%, respectively, translating to 854 and 450 excess AF presentations annually [13]. The NO_2_ threshold identified in that analysis, 16.5 ppb corresponds to approximately 31 µg/m^3^ at 25 °C and 1013 hPa and therefore exceeds, rather than falls below, the WHO 2021 24 h air quality guideline level for NO_2_ of 25 µg/m^3^ [14]. The concern about regulatory adequacy nevertheless survives, on stronger grounds. In an individual-level, time-stratified case-crossover analysis of 190,115 patients presenting with symptomatic arrhythmia across 322 Chinese cities, Xue et al. found approximately linear exposure–response relationships for PM_2.5_, NO_2_, SO_2_ and CO with no discernible concentration threshold [15], and the most recent umbrella review of PM_2.5_ and cardiovascular outcomes, appraising 38 systematic reviews and meta-analyses with AMSTAR 2 and ROBIS, reports elevated cardiovascular risk at concentrations below the WHO annual guideline level of 5 µg/m^3^ and below the corresponding United States and Indian national standards [16]. It is the absence of a demonstrable threshold, rather than the position of any single study’s cut-point relative to a guideline, that challenges the logic of threshold-based regulation for arrhythmic endpoints.

Furthermore, increases in ambient PM_2.5_, CO, NO_2_, and ozone concentrations have all been linked to AF events detected by CIEDs, increased emergency admissions, higher stroke rates in anticoagulated patients, and elevated mortality in individuals with known AF [17]. Beyond AF, short-term PM_2.5_ exposure was associated with increased ventricular premature complex burden in a Taiwanese cohort without structural heart disease, pointing to a shared arrhythmogenic mechanism across supraventricular and ventricular substrates that likely involves systemic oxidative stress and autonomic dysregulation [18].

Evidence that ambient pollution acts across the supraventricular spectrum, and not on atrial fibrillation alone, has accumulated rapidly. In 442,386 UK Biobank participants followed prospectively, Zhou et al. found that a composite air-pollution score combining PM_2.5_, PM_10_, NO_2_ and NO_X_ was associated with incident supraventricular tachycardia at a hazard ratio of 2.63 per unit increment, an estimate substantially larger than the corresponding hazard ratio of 1.45 for atrial fibrillation, with stronger associations in women, in older participants and in those with obesity or cardiometabolic comorbidity [19]. At shorter timescales, the analysis by Xue et al. of 190,115 individual arrhythmia onsets found that an interquartile-range increase in PM_2.5_, NO_2_, SO_2_ or CO over the preceding 24 h raised the odds of supraventricular tachycardia onset by between 3.4% and 8.9%, and that ozone exposure was associated with a 3.4% increase [15].

The biological plausibility for pollution-triggered SVTs encompasses several non-mutually exclusive pathways: (1) direct particulate entry into the circulation triggering inflammatory cytokine release; (2) autonomic activation via pulmonary irritant receptors increasing cardiac sympathetic tone; (3) endothelial dysfunction and elevated right atrial pressure predisposing to ectopic activity; and (4), most relevant to this review, epigenetic reprogramming of atrial cells under sustained oxidative and inflammatory stress [13,20]. Of these four pathways, the first three are supported by direct human physiological data, while the fourth is currently supported only by evidence obtained in circulating cells [20] and by inference from atrial tissue studies in which exposure was not measured.

**Table 1:** Quantitative associations between environmental exposures and SVT.

Exposure	Study, Design and Size	Outcome	Effect Estimate (95% CI)	Ref.
PM_2.5_, short-term	Liu et al.; case-crossover, CIED-detected episodes, Beijing	AF occurrence	+3.8% odds per 10 µg/m^3^ (1.4–6.2)	[11]
PM_2.5,_ 48–96 h lag	Dahlquist et al.; case-crossover, intracardiac devices, Sweden	AF episodes	Significant association at concentrations below WHO guideline levels	[12]
NO_2_ ≥ 16.5 ppb (≈31 µg/m^3^)	Dawson et al.; time-series, 82,575 ED presentations, Australia, 7 years	AF ED presentation	Population-attributable fraction 7.24%; 854 excess presentations/year	[13]
PM_2.5_ ≥ 57.7 µg/m^3^	Dawson et al. (as above)	AF ED presentation	Population-attributable fraction 3.81%; 450 excess presentations/year	[13]
PM_2.5_, short-term	Matteucci et al.; meta-analysis, 4 studies	AF	RR 1.045 (1.025–1.066); I^2^ = 0.66%	[21]
PM_2.5_, long-term	Matteucci et al.; meta-analysis	AF	Pooled ratio 1.077 (1.002–1.158); I^2^ = 99.9%; not interpretable quantitatively	[21]
PM_2.5_, any size, short-term	Sethi et al.; umbrella review, 38 SR/MA	Arrhythmia (composite)	RR 1.015 (1.006–1.024)	[16]
Composite air-pollution score, long-term	Zhou et al.; prospective cohort, UK Biobank, *n* = 442,386	Supraventricular tachycardia	HR 2.63 per unit increment	[19]
Composite air-pollution score, long-term	Zhou et al. (as above)	Atrial fibrillation	HR 1.45 per unit increment	[19]
PM_2.5_,/NO_2_/SO_2_/CO, 0–24 h	Xue et al.; individual-level case-crossover, *n* = 190,115, 322 cities	Supraventricular tachycardia onset	+3.4% to +8.9% odds per IQR increase	[15]
PM_10_, 0–24 h	Xue et al. (as above)	Supraventricular tachycardia onset	+5.4% odds per IQR increase	[15]
Ozone, 0–24 h	Xue et al. (as above)	Supraventricular tachycardia onset	+3.4% odds per IQR increase	[15]
PM_2.5_, short-term	Tsai et al.; cohort, consecutive Holter recordings, Taiwan	Ventricular premature complex burden	Significant positive association	[18]
Cigarette smoking	Joehanes et al.; EWAS meta-analysis	Blood DNA methylation	2623 differentially methylated CpG sites; 185 persisting after cessation	[22]
Smoking-related methylation at cg25313468 (REST)	Cao et al.; Mendelian randomization with colocalization	AF and coronary disease	Causal pathway supported	[23]
Diesel exhaust, controlled exposure	Jiang et al.; double-blind crossover, *n* = 16	Mononuclear-cell DNA methylation	2827 CpG sites altered vs. filtered air, including a site in the miR-21 locus	[20]
Alcohol, 12 g/day	Csengeri et al.; pooled European cohort, *n* = 107,845, ~14 years	Incident AF	+16% risk per standard drink/day	[24]
Alcohol ≥ 6 drinks/day	Klatsky; systematic analysis	AF, flutter, SVT, PACs	Approximately twofold risk versus lighter drinkers	[25]
Intravenous alcohol to BAC 0.08%	Marcus et al.; randomized, double-blind, placebo-controlled	Pulmonary vein effective refractory period	Acute shortening	[26]
Epigenetic age acceleration (DNAm GrimAge)	Roberts et al.; 3 cohorts, *n* = 5600, 905 events	Incident AF	HR 1.19 per 5 years (1.09–1.31); Mendelian randomization null	[27]
Genetic versus environmental partition	Frimodt-Møller et al.; twin study, 32,324 pairs	Supraventricular tachycardia	35% additive genetic, 65% unique environmental	[28]

### 3.2. Tobacco Smoke and Occupational Pollutants

Cigarette smoking represents the most characterized environmental modulator of the cardiac epigenome. A landmark meta-analysis of genome-wide DNA methylation identified 2623 differentially methylated CpG sites in blood from current smokers versus never-smokers, annotated to 1405 genes, many of which are directly implicated in pulmonary function, cancer, inflammatory disease, and cardiovascular disease [22]. Critically, 185 CpG sites remained differentially methylated years after smoking cessation, demonstrating that tobacco imprints an epigenetic scar that persists long after behavioral change, which poses a sobering reminder of the inadequacy of cessation alone as a restorative strategy.

Moreover, Cao et al. used Mendelian randomization and colocalization analysis to demonstrate causal pathways through which smoking-related DNA methylation at specific CpG sites increases the risk of AF [23]. The site cg25313468, located in the TSS1500 region of the REST (RE1-silencing transcription factor) gene, was simultaneously associated with AF, coronary atherosclerosis, coronary heart disease, and myocardial infarction, highlighting how a single epigenetically dysregulated hub gene can mediate pleiotropic cardiovascular risk. REST is known to repress neuronal gene expression in cardiac cells, and its dysregulation has plausible consequences for conduction system function [23].

The integration of methylome and transcriptome data by Maas et al. in the Rotterdam Study extended these findings by demonstrating that specific smoking-related CpG changes were functionally linked to altered gene expression and, subsequently, to cardiometabolic traits, thereby mapping the causal chain from tobacco exposure to cardiac risk [29]. Occupational exposures to fine particles, biomass combustion products, and organochlorine compounds, which are common in low- and middle-income country settings, are likely to exert analogous epigenetic effects, though data specific to cardiac arrhythmias in these contexts remain insufficient.

### 3.3. Obesity, Physical Inactivity, and Metabolic Stress

Obesity and its metabolic consequences constitute one of the most powerful modifiable risk factors for AF and SVTs more broadly. Epidemiological evidence now conclusively links adiposity to incident AF, with mechanisms including increased left atrial volume, elevated inflammatory cytokines, altered autonomic tone, and structural atrial remodeling [30]. Karakasis et al. identified epicardial adipose tissue (EAT) as a particularly important mediator of obesity–AF linkage: as a visceral fat depot contiguous with the myocardium and sharing its microvasculature, EAT functions as a cardio-proximal immunometabolic interface, deploying paracrine fibroinflammatory signals directly into atrial tissue [31].

What makes obesity particularly relevant to an epigenomic framework is that adiposity is associated with global and locus-specific DNA methylation changes affecting metabolic genes, and that weight reduction only partially reverses these obesity-imprinted epigenetic programs across subcutaneous, visceral, and epicardial depots [31,32]. This epigenetic memory of obesity means that cardiovascular risk can persist even after metabolic normalization, which has sobering implications for the durability of weight-loss interventions as AF prevention strategies. Epigenetic modifications in obesity relevant to cardiac disease include altered methylation at genes encoding adipokines, inflammatory mediators, and calcium-handling proteins, all of which can modulate atrial electrophysiology.

Physical inactivity amplifies the metabolic and inflammatory burden of obesity, and conversely, regular exercise exerts measurable epigenomic benefits. Studies in the AF context have found that moderate-intensity exercise reduces both AF burden and inflammatory biomarkers [33]. The specific epigenetic mechanisms underlying exercise-related atrial protection have not been characterized in human atrial tissue. Data from other tissues suggest that physical activity may modulate methylation at promoters of pro-inflammatory and pro-fibrotic genes, which could counteract the epigenetic drift imposed by sedentary, obesogenic environments. The appropriate exercise dosage for SVT prevention remains debated, as extreme endurance athletic exposure (“athlete’s AF”) may paradoxically promote a vagally mediated fibrotic substrate, that is a compelling example of how the same environmental exposure exerts differential epigenetic effects depending on context and intensity [33].

### 3.4. Alcohol Consumption

The association between alcohol consumption and SVTs, particularly the “holiday heart syndrome” of AF emerging after binge drinking, has been recognized for decades. Klatsky, in a systematic analysis, documented a twofold higher risk of AF, atrial flutter, supraventricular tachycardia, and premature atrial contractions in individuals consuming six or more drinks per day compared with lighter drinkers, noting that the risk extended across the spectrum of supraventricular arrhythmias rather than being exclusive to AF [25].

More recent work has refined the dose–response relationship. Csengeri et al., in a pooled European cohort of 107,845 individuals followed for nearly 14 years, found a non-linear but consistently positive association between alcohol consumption and incident AF, with even one standard drink (12 g) per day associated with a 16% increase in AF risk, an effect not fully explained by cardiac biomarker concentrations or comorbid heart failure [24]. A randomized, double-blind, placebo-controlled trial by Marcus et al. using intravenous alcohol titrated to a blood alcohol concentration of 0.08% demonstrated acute shortening of pulmonary vein effective refractory periods, providing the first experimental mechanistic bridge between a lifestyle exposure and a direct atrial electrophysiological abnormality in humans [26].

The mechanisms by which chronic alcohol consumption induces SVT substrates include atrial dilatation and elevated pressures, fibrosis formation, autonomic imbalance, and contributions to comorbid AF risk factors (obesity, hypertension, sleep-disordered breathing) [34]. Ethanol also directly modulates multiple ion channel targets in cardiomyocytes, including I_na_, I_t_^o^, and gap junction conductance, in a concentration-dependent manner [35]. From an epigenetic standpoint, Wu et al. reviewed evidence that ethanol-triggered epigenetic modifications, specifically DNA methylation changes and histone deacetylation, mediate the cardiac remodeling phenotype of alcoholic cardiomyopathy and are detectable in blood, positioning them as potential diagnostic and prognostic biomarkers [36].

### 3.5. Psychosocial Stress and Dietary Patterns

Psychosocial stress activates hypothalamic–pituitary–adrenal and sympathoadrenal axes, generating a hormonal milieu, characterized by elevated cortisol and catecholamines, that has well-established arrhythmogenic consequences. Psychological stressors can trigger acute AF episodes through sympathetic activation, adrenergic receptor sensitization, and heightened ectopic atrial firing, while chronic stress may contribute to a persistent arrhythmogenic substrate through inflammatory and metabolic intermediaries [37].

Dietary patterns modulate SVT risk primarily through their downstream effects on inflammatory load, metabolic status, and the gut microbiome. Mediterranean dietary adherence has been associated with reduced AF incidence in several observational studies [38], plausibly through anti-inflammatory polyphenols and omega-3 fatty acids that modulate NF-κB signaling and inflammatory gene methylation patterns. Conversely, ultra-processed food intake, which is increasingly prevalent in urbanizing populations, promotes insulin resistance, visceral adiposity, and systemic inflammation, each of which is associated with epigenetic pro-arrhythmic remodeling.

## 4. Mechanistic Epigenetics: DNA Methylation, Histone Modifications, and Non-Coding RNAS in SVT Substrates

### 4.1. The Epigenetic Architecture of the Atrium

The eukaryotic genome is packaged into chromatin, where DNA is wrapped around histone octamers to form nucleosomes. Epigenetic regulation operates at multiple levels of this architecture: (1) DNA methylation at CpG dinucleotides; (2) covalent histone tail modifications including acetylation, methylation, phosphorylation, and ubiquitination; and (3) non-coding RNAs that regulate gene expression post-transcriptionally or guide chromatin-modifying complexes to specific genomic targets [6]. These layers do not function in isolation; chromatin remodeling enzymes, histone methylation patterns, and DNA methylation are interdependent, with disruption in one invariably propagating to others [39].

Mapping the epigenomic landscape of the human left atrium has been a critical advance. Hall et al. profiled seven histone post-translational modifications in left atrial tissue from individuals without structural heart disease and constructed a 21-state model encompassing promoters, enhancers, and repressed regions [40]. This resource, intersected with GWAS loci and chromatin interaction data, demonstrated that over 15,000 left atrium-specific enhancers, defined by homeobox family transcription factor motifs, can be annotated for AF susceptibility variants, and that a gene interaction network dominated by PITX2, NKX2-5, TBX5, and ZFHX3 emerges from chromatin conformation analysis. This work established that a substantial fraction of AF genetic risk operates through the epigenetic landscape rather than through protein-coding mutations, a finding with profound implications for understanding how environmental exposures might converge with inherited risk.

### 4.2. DNA Methylation in Ion Channel Genes, Gap Junction Proteins, and Fibrosis Regulators

DNA methylation, the covalent addition of a methyl group to the 5′ position of cytosine in CpG dinucleotides, is the most extensively characterized epigenetic mechanism in cardiovascular disease. Enzymatic methylation by DNMT1 (maintenance methylation), DNMT3A, and DNMT3B (de novo methylation) and active demethylation mediated by the TET family of dioxygenases create a dynamic methylome that is highly responsive to environmental signals [41].

In AF, global DNA methylation is significantly elevated in atrial tissue compared to sinus rhythm controls, accompanied by hypermethylation of specific loci including the natriuretic peptide receptor-A (NPRA) gene promoter [42]. Suppression of NPRA, a receptor that modulates intracardiac pressure and fibrotic signaling, by promoter hypermethylation may impair endogenous anti-fibrotic defenses and thus contribute to the structural substrate of persistent AF. Importantly, DNMT3B was implicated as the primary driver of these dysregulations, suggesting a specific enzymatic target for intervention.

Of particular electrophysiological relevance is methylation-dependent silencing of ion channel genes. SCN5A, encoding the cardiac sodium channel Nav1.5, and the potassium channel genes KCNQ1, KCNH2, and KCNJ2 are subject to CpG methylation-dependent transcriptional regulation [43]. Reduced Nav1.5 expression arising from SCN5A hypermethylation would be predicted to slow conduction velocity in atrial tissue and thereby to favor re-entry [43]. Similarly, altered methylation of connexin-encoding genes (GJA1, encoding Cx43; GJA5, encoding Cx40) modulates gap junctional coupling between atrial cardiomyocytes, and connexin downregulation has been documented in AF atria [43]. Discontinuous conduction from gap junction remodeling creates the anisotropic substrate that facilitates re-entrant wavelets.

The TGF-β/SMAD signaling axis, which orchestrates atrial fibrosis through fibroblast activation, is itself subject to multi-layered epigenetic regulation. TGF-β1 promotes atrial fibrosis by activating SMAD2/3 transcription factors that drive collagen deposition; this process is amplified when competing anti-fibrotic pathways, such as BMP signaling, are suppressed by promoter methylation [7]. Environmental stressors, including PM_2.5_ and oxidative metabolites from tobacco smoke, are known to activate TGF-β signaling through ROS-mediated pathways, providing a molecular bridge between pollution exposure and structural atrial remodeling. Progressive atrial fibrosis creates a substrate of slow, discontinuous conduction in which multiple re-entrant circuits can be sustained, which is particularly relevant to AF and focal atrial tachycardias.

### 4.3. Histone Modifications and Chromatin Remodeling Under Oxidative and Inflammatory Stress

Post-translational modifications of histone tails profoundly regulate chromatin accessibility and gene expression. Acetylation of lysine residues, catalyzed by histone acetyltransferases (HATs) and reversed by histone deacetylases (HDACs), generally promotes open, transcriptionally active chromatin, while methylation marks can be activating (H3K4me3 at active promoters) or repressive (H3K27me3 deposited by the PRC2 complex, H3K9me3 at heterochromatin) [39,41].

Critically, cardiac injury activates pathological histone modification cascades in cardiomyocytes and fibroblasts [39]. Three modifications are mechanistically relevant to the SVT substrate. Reduced H3K4me3 at calcium-handling gene promoters, particularly SERCA2A/ATP2A2, impairs calcium sequestration into the sarcoplasmic reticulum and predisposes to delayed afterdepolarizations and triggered arrhythmias. Increased H3K27me3 deposition by EZH2 at atrial repressor loci can dysregulate the atrial gene program. Aberrant HDAC activation drives pro-fibrotic gene expression in cardiac fibroblasts by deacetylating anti-fibrotic transcription factors [39].

Sirtuins, NAD-dependent class III HDACs, warrant specific attention in the context of environmental and metabolic stress. SIRT1 deacetylates and activates FOXO transcription factors and NF-κB targets, providing epigenetic-level regulation of the inflammatory response. SIRT3 is a critical mitochondrial deacetylase that prevents ROS accumulation and its downregulation under conditions of metabolic excess (obesity, high-fat diet) directly increases mitochondrial ROS production, which can trigger atrial oxidative stress-mediated fibrosis [7]. Ethanol metabolism generates acetaldehyde, which broadly inhibits HDAC activity through Zn^2+^ chelation, leading to global histone hyperacetylation with dysregulated pro-inflammatory gene expression. This observation may partly explain why habitual alcohol consumption provokes a persistent epigenetic pro-arrhythmic state that outlasts any single drinking episode [36].

Particulate matter exposure activates NF-κB signaling and generates systemic pro-inflammatory cytokines, and controlled human exposure to diesel exhaust perturbs DNA methylation at loci enriched for NF-κB and protein kinase pathway genes in circulating mononuclear cells [20]. Whether the resulting cytokine milieu promotes H3K27 acetylation at inflammatory gene loci in atrial cardiomyocytes has not been tested directly, and we present it here as a hypothesis rather than a finding. Its appeal is that H3K27ac is the canonical enhancer-activating mark, and that AF-associated variants are concentrated in left atrium-specific enhancers whose activity is defined by that mark [40]. If the hypothesis is correct, pollution exposure would reshape the atrial enhancer landscape in a manner that amplifies the transcriptional response to subsequent inflammatory stimuli.

### 4.4. The Role of Non-Coding RNAs in Environmental Responsiveness and Arrhythmogenic Circuits

MicroRNAs (miRNAs) are small (~22 nucleotide) non-coding RNAs that silence gene expression post-transcriptionally by binding to the 3′-UTR of target mRNAs. Long non-coding RNAs (lncRNAs), operationally defined as non-coding transcripts >200 nucleotides, regulate gene expression through diverse mechanisms including chromatin scaffolding, transcription factor sequestration, and competing endogenous RNA (ceRNA) sponging of miRNAs [4].

The miRNA landscape in AF is complex and incompletely cataloged, but a systematic review and meta-analysis of 40 studies by Shen et al. identified 51 consistently dysregulated miRNAs in AF, with the most robustly downregulated being miR-1-5p and the most upregulated being miR-223-3p [43]. Of clinical interest, Menezes Júnior et al.’s meta-analysis confirmed significant associations between circulating levels of miR-133a, miR-150, miR-21, miR-4443, miR-4798, and miR-20a-5p and AF status, providing proof of concept for non-coding RNA-based biomarker development [44].

Several miRNAs merit detailed consideration. miR-1, which is the most abundant cardiac miRNA, is downregulated in AF and targets KCNJ2 (encoding Kir2.1) and KCNE1. Downregulation of miR-1 leads to upregulation of the inward rectifier potassium current and consequent shortening of the atrial action potential duration, facilitating re-entry [45]. miR-21, which is robustly upregulated in AF, targets SPRY1, a negative regulator of ERK signaling, in cardiac fibroblasts; the resulting augmentation of ERK-MAP kinase activity enhances fibroblast survival and paracrine growth-factor secretion and promotes interstitial fibrosis, and antagomir-mediated silencing of miR-21 reverses these effects in a murine pressure-overload model [46]. miR-133, which is frequently downregulated in AF, normally represses RhoA, MAPK, and TGF-β/SMAD signaling and its loss further amplifies the pro-fibrotic cascade [47]. miR-29 family members target collagen genes (COL1A1, COL3A1) and are consistently downregulated in fibrotic atrial tissue [48].

Several of these miRNAs are demonstrably environmentally responsive, and the strongest evidence comes from a controlled human exposure. In a double-blind crossover study, Jiang et al. exposed sixteen non-smoking asthmatic participants to diesel exhaust and to filtered air and found methylation changes at 2827 CpG sites following diesel exposure but not following filtered air, with affected sites enriched for protein kinase and NF-κB pathway genes and including a site within the miR-21 locus and a site in GSTP1 [20]. The randomized, sham-controlled, within-participant design establishes temporality and constrains confounding to a degree that observational exposure studies cannot, and it is the strongest available evidence that a defined air-pollution exposure perturbs the epigenetic regulation of a cardiac-relevant miRNA in humans. It does not, however, demonstrate remodeling in atrial tissue. No human study has profiled atrial miRNA expression against measured pollutant exposure, principally because atrial sampling is confined to cardiac surgery and is not undertaken in exposure-defined cohorts.

Long non-coding RNAs represent an equally rich, if less well-characterized, dimension of non-coding RNA biology in AF. MIAT (myocardial infarction-associated transcript) regulates atrial fibrosis through sponging of miR-133a, removing a brake on fibroblast-activating TGF-β signaling [45]. NRON (non-coding repressor of NFAT) normally sequesters the NFAT transcription factor in the cytoplasm and its downregulation in AF allows nuclear NFAT translocation and subsequent transcription of pro-hypertrophic and pro-fibrotic genes [45]. PCAT1 promotes atrial fibroblast proliferation by targeting TGF-β1. These lncRNAs constitute, in effect, regulatory nodes at which environmental signals, transmitted through transcription factor activation and chromatin remodeling, converge to amplify the fibrotic and electrical remodeling characteristic of advanced AF. Table 2 summarizes environmental exposures and their principal epigenetic mechanisms, key molecular targets and downstream electrophysiological or structural consequences.

**Table 2:** Environmental exposures, principal epigenetic mechanisms, key molecular targets and downstream electrophysiological or structural consequence.

Exposure	Principal Epigenetic Mechanism	Key Molecular Targets	Downstream Electrophysiological or Structural Consequence	Ref.
Traffic-related pollution (diesel exhaust)	DNA methylation change at inflammatory and kinase-pathway loci; miRNA locus methylation	NF-κB pathway genes, *GSTP1*, miR-21 locus	Systemic inflammatory priming; hypothesized atrial enhancer remodeling	[20]
PM_2.5_ and traffic-related pollution	ROS-mediated TGF-β activation	TGF-β1, SMAD2/3, *COL1A1*, *COL3A1*	Atrial fibrosis; slow, discontinuous conduction	[7]
PM_2.5_ and traffic-related pollution	Hypothesized H3K27 acetylation at inflammatory enhancers	Left atrium-specific enhancer repertoire	Amplified transcriptional response to subsequent inflammatory stimuli	[40]
Tobacco smoke	Locus-specific DNA methylation, persisting after cessation	2623 CpG sites/1405 genes; *AHRR* cg05575921, *F2RL3*	Cardiometabolic risk; exposure biomarker	[22,29]
Tobacco smoke	Methylation at cg25313468 in the REST TSS1500 region	REST	AF, coronary atherosclerosis, myocardial infarction; MR-supported causal pathway	[23]
Obesity and epicardial adipose tissue	Global and locus-specific methylation change, incompletely reversed by weight loss	Adipokine, inflammatory and calcium-handling genes	Paracrine fibroinflammatory signaling into atrial tissue; atrial remodeling	[31,32]
Obesity and metabolic excess	SIRT3 downregulation; mitochondrial protein hyperacetylation	SIRT3, mitochondrial ROS scavenging	Oxidative stress-mediated atrial fibrosis	[7]
Alcohol	Acetaldehyde-mediated HDAC inhibition via Zn^2+^ chelation; global histone hyperacetylation	HDAC family; pro-inflammatory gene loci	Persistent pro-arrhythmic transcriptional state outlasting the exposure	[36]
Alcohol, acute	Non-epigenetic; direct ion-channel modulation	I_Na, I_to, gap junction conductance	Pulmonary vein effective refractory period shortening	[26,35]
Physical inactivity	Hypothesized methylation change at pro-inflammatory and pro-fibrotic promoters	Not characterized in atrium	Hypothesized loss of exercise-associated atrial protection	[33]
Psychosocial stress	HPA and sympathoadrenal activation; epigenetic intermediates not characterized in atrium	Adrenergic receptor sensitization	Acute AF triggering; hypothesized chronic substrate	[37]
Diet, Mediterranean pattern	Hypothesized modulation of NF-κB signaling and inflammatory gene methylation	NF-κB pathway	Reduced AF incidence in observational and trial data	[38]
Aging under environmental load	Methylation-based epigenetic age acceleration	DNAm GrimAge, DNAm PhenoAge	Incident AF (observational HR 1.19 per 5 y); Mendelian randomization null	[27]
Composite pollution exposure	Not measured	Not measured	Incident supraventricular tachycardia (HR 2.63)	[19]

## 5. Evidence from CVD Applied to SVTs: AF as a Mechanistic Template and Its Limits

### 5.1. Atrial Fibrillation: The Epigenomic Prototype

Atrial fibrillation represents the most systematically studied SVT from an epigenomic perspective [49]. Vinciguerra, Dobrev, and Nattel, in their 2024 Lancet Regional Health Europe review, provide the most current synthesis of AF genetic and epigenetic regulatory networks, emphasizing that the vast majority of AF-associated GWAS variants lie within non-coding regulatory regions where their functional effects are mediated through alterations in transcription factor binding and chromatin state rather than direct protein-coding changes [2]. This non-coding architecture means that the AF epigenome is inherently more responsive to environmental perturbation than a simple Mendelian model would predict: enhancers and promoters whose activity is shaped by histone marks and DNA methylation are precisely the regulatory elements most amenable to environmental remodeling.

Donate Puertas et al. further outlined how atrial remodeling encompasses ion channel dysfunction, calcium handling abnormalities, and structural fibrosis, each with distinct epigenetic mediators, and argued that the convergence of these epigenetic mechanisms on a common fibroinflammatory endpoint may explain why AF becomes self-sustaining: “AF begets AF”, not merely through mechanical stretch and calcium overload, but through epigenetic stabilization of the remodeled atrial phenotype [49]. Karakasis et al., in 2025, cataloged systemic stressors, such as aging, obesity, diabetes, hypertension, hypoxia, and alcohol, as epigenetic reprogrammers that collectively advance atrial cardiomyopathy, and noted that circulating miRNAs are emerging as diagnostic and prognostic markers of this process [50].

Epigenomic profiling of human left atrial tissue has begun to move the field from associations to functional gene networks. Hall et al. demonstrated that differential histone modifications between atrial regions predict transcription factor binding and gene expression signatures relevant to AF susceptibility, and that AF-GWAS loci cluster within LA-specific enhancers controlled by the PITX2/TBX5 transcriptional network [40]. Van Ouwerkerk et al. integrated GWAS loci with epigenetic state annotations and transcriptomic data to define a transcriptional regulatory network for AF, showing how dose-sensitive transcription factors at GWAS loci act through epigenetically regulated enhancers to control the expression of effector genes governing ion channel currents, intercellular coupling, and structural homeostasis [51].

### 5.2. From AF to Other SVTs: Evidence, Extrapolation, and Epistemic Caution

AVNRT, the most common paroxysmal SVT in adults, arises from functional (and likely anatomical) duality within the compact AV node, enabling re-entrant excitation through separate fast and slow pathways. AVRT involves re-entry through one or more accessory atrioventricular connections. Focal atrial tachycardias originate from a discrete site of enhanced automaticity, triggered activity, or micro re-entry. These mechanisms are mechanistically distinct from the multiple-wavelet re-entry and rotor dynamics that sustain AF [52,53,54].

The genetic architecture of AVNRT and AVRT is less densely mapped than that of atrial fibrillation, but it is no longer unresolved, and the recent literature changes the terms on which extrapolation from AF must be justified. In a nationwide study of 32,324 Danish twin pairs, of which 663 contained at least one member with a supraventricular tachycardia diagnosis, Frimodt-Møller et al. found the risk of SVT in a co-twin following the index twin’s diagnosis to be substantially higher in monozygotic than in dizygotic pairs (hazard ratio 3.61, 95% CI 1.35–9.63; 3.30, 1.24–8.89 after adjustment for age and sex), with proband-wise concordance of 9% versus 3%. Biometrical modeling attributed approximately 35% of SVT risk to additive genetic factors and 65% to unique environmental factors [28]. That partition is the quantitative warrant for the present review: across the SVT spectrum, the environmental component is roughly twice the genetic one, and an account of susceptibility framed exclusively in terms of inherited variation is incomplete by construction.

Specific susceptibility loci have now been identified. Andreasen et al. reported common variants close to TTN, NKX2-5 and MYH6 in association with AVNRT [55], and a subsequent multi-ancestry meta-analysis of genome-wide association studies by Weng et al., comprising 2384 AVNRT cases with 106,489 referents and 2811 accessory-pathway or AVRT cases with 1,483,093 referents, reported genome-wide significant loci for both phenotypes. Across the two studies the AVNRT signals implicate the cardiac developmental transcription factor NKX2-5 and the sarcomeric genes TTN and MYH6, while the accessory-pathway signals implicate the sodium channel genes SCN5A and SCN10A and the TTN-CCDC141 region [55,56]; a transcriptome-wide association analysis supported a relationship between reduced predicted cardiac NKX2-5 expression and AVNRT risk [56]. Two of the suggestive AVNRT signals reported in that analysis bear directly on the framework advanced here. The first is PRRX1, which is also an established atrial fibrillation susceptibility locus and appears among the developmental transcription factors; its presence in both phenotypes is the first locus-level evidence that AF and AVNRT share any part of their genetic architecture. The second is DPF3, which encodes a chromatin reader within the BAF remodeling complex expressed in cardiac and skeletal muscle [56]. To our knowledge this is the first genetic signal implicating the epigenetic machinery itself in a non-AF supraventricular arrhythmia. Both are suggestive rather than genome-wide significant associations, and neither has been functionally validated.

These findings should not be over-read. The AV node and accessory pathways express ion channel, connexin and transcription factor profiles distinct from those of working atrial myocardium, and the epigenomic response of these specialized tissues to environmental stressors may diverge substantially from what has been documented in the atrial wall. No methylation, chromatin-accessibility or histone-modification data have been reported for AV nodal or accessory-pathway tissue in any exposure context, and the participants in the AVNRT and accessory-pathway meta-analyses were 98.6% and 84.6% of European ancestry respectively [56], so even the genetic findings are of uncertain generalizability.

First, inflammatory states, which are established modulators of both epigenetic programming and AF risk, have been implicated in AV nodal dysfunction and enhanced automaticity in focal atrial tachycardias. Second, connexin remodeling, which impairs intercellular coupling in AF, also characterizes conduction system pathologies relevant to AVNRT substrates. Third, miRNA signatures of electrical and structural remodeling (downregulation of miR-1, miR-133) are not AF-specific but reflect general responses of atrial myocardium to stress that are also plausibly operative in the specialized atrial and nodal tissue of AVNRT and AVRT substrates. Fourth, clinical phenotyping now suggests that AVNRT substrate is not uniformly congenital. In a two-step cluster analysis of 305 patients undergoing AVNRT ablation, Hasdemir et al. identified three mechanistically distinct phenotypes, one of which, comprising 44 patients, was characterized by markedly later onset (mean 53.5 years versus 24.7 years in the largest cluster), universal antecedent type 2 diabetes, high prevalence of antecedent structural heart disease, and substantially higher rates of drug-induced pro-arrhythmia, atrioventricular conduction impairment and aborted ablation [57]. The existence of a metabolic-structural AVNRT phenotype is consistent with an acquired, exposure-modifiable contribution to nodal substrate.

The appropriate conclusion is that AF-derived epigenomic insights constitute a plausibility framework and a set of testable hypotheses for AVNRT, AVRT, and focal atrial tachycardias, but not a validated mechanistic model (Table 3). The field urgently requires studies specifically designed to characterize the epigenomic states of AV nodal tissue and accessory pathways in response to the same environmental stressors that have been studied in AF, an agenda that is technically demanding but not impossible with contemporary surgical and catheter-based tissue acquisition approaches.

**Table 3:** SVT subtypes: electrophysiological substrate, genetic architecture and epigenomic data availability.

	Atrial Fibrillation	AVNRT	AVRT/Accessory Pathway	Focal Atrial Tachycardia
Electrophysiological substrate	Multiple-wavelet re-entry, rotors, pulmonary vein triggers, diffuse atrial fibrosis	Functional and probable anatomical duality of the compact AV node; fast and slow pathways	Re-entry through one or more accessory atrioventricular connections	Discrete focus of enhanced automaticity, triggered activity or micro-re-entry
Tissue of interest	Left atrial wall, pulmonary vein ostia, right atrial appendage	Compact AV node, triangle of Koch	Accessory pathway myocardium, AV groove	Variable; crista terminalis, pulmonary vein ostia, tricuspid annulus
GWAS loci, genome-wide significant	525 loci; *PITX2*, *ZFHX3*, *TBX5*, *PRRX1*, *NEURL1*, *HAND2*, *SCN5A*, *KCNN3*, *TTN* [5,58,59]	2 genome-wide significant loci; signals across the two studies implicate *NKX2-5*, *TTN* and *MYH6* [55,56]	3 genome-wide significant loci implicating *SCN5A*, *SCN10A* and the *TTN*-*CCDC141* region [56]	Underpowered; no established loci [56]
Suggestive loci of epigenetic relevance	Enhancer-resident variants throughout; >15,000 LA-specific enhancers annotated [40]	*PRRX1* (shared with AF); *DPF3* (BAF-complex chromatin reader) [56]	*ANKRD28*, *NEDD9* [56]	None reported
Cases in largest genetic analysis	168,007 [5]	2384 [56]	2811 [56]	Not separately analyzed
Ancestry composition of that analysis	Multi-ancestry [5]	98.6% European [56]	84.6% European [56]	—
DNA methylation data in the relevant tissue	Yes; global hypermethylation, *NPRA* promoter, ion channel and connexin loci [42,45]	None reported	None reported	None reported
Histone modification/chromatin accessibility data	Yes; 7 marks profiled, 21-state chromatin model of human left atrium [40]	None reported	None reported	None reported
Non-coding RNA data	Yes; 51 consistently dysregulated miRNAs; validated circulating panels [43,44]	None reported	None reported	None reported

### 5.3. Methodological Limitations

Several methodological challenges merit explicit acknowledgement. First, most human epigenetic data in AF derives from right atrial appendage biopsies obtained at cardiac surgery, which are not representative of all atrial regions, the left atrium (the dominant site of AF remodeling), the pulmonary vein ostia, or the AV node. Second, epigenome-wide studies typically use mixed cell populations (cardiomyocytes, fibroblasts, endothelial cells, and immune infiltrate) obscuring the cell-type-specific origins of observed epigenetic changes. Single-cell epigenomics can resolve this limitation but has not yet been systematically applied to human SVT tissue [2,49].

Third, causality is difficult to establish in human epigenomic studies. Differentially methylated regions observed in AF tissue could represent causal drivers of arrhythmogenesis, downstream consequences of the arrhythmia itself, or bystander changes related to comorbidities. Mendelian randomization approaches using genetic variants as instrumental variables can partially disentangle these relationships in the context of specific exposures, as demonstrated for smoking [23], but genome-scale Mendelian randomization for air pollution and epigenetic outcomes in atrial tissue remains technically and statistically challenging.

Fourth, the inference from peripheral-blood methylation to atrial tissue state is weaker than it is usually presented to be. Ma et al., using paired peripheral blood leucocyte, atrial and arterial samples collected in a study of post-operative atrial fibrillation, reported an unadjusted cross-tissue correlation between blood and atrium of R^2^ ≈ 0.39 at CpG sites variable in the target tissue; a locus-specific statistical recalibration raised this to R^2^ ≈ 0.95, but only for such sites and only after training a model on paired samples [60]. In a smaller comparison across blood, buccal cells and right ventricular myocardium in infants, approximately 17% of interrogated CpG sites differed significantly between blood and myocardium, whereas buccal tissue differed at only 1.3% of sites [61]. Blood is therefore a usable but non-interchangeable proxy: defensible as an exposure biomarker, defensible as a recalibrated surrogate at pre-specified loci, and not defensible as an unmodelled read-out of atrial methylation state. Infante et al. illustrate the productive middle path, deriving candidate AF biomarkers from CD4^+^ T-cell methylomes while explicitly designating the resulting genes indirect, epigenetically sensitive markers rather than reporters of atrial tissue [62].

Fifth, sex and genetic ancestry modify both exposure response and epigenetic architecture, and the literature synthesized here is unbalanced with respect to both. Women exhibit greater atrial fibrosis, longer P-wave durations, higher post-ablation recurrence and distinct hormonal modulation of atrial electrophysiology [63], and the UK Biobank analysis found stronger pollution–arrhythmia associations in women, in older participants and in those with cardiometabolic comorbidity [19]. Almost none of the mechanistic epigenomic studies cited here were powered for sex-stratified analysis. Similarly, although the most recent cross-population AF GWAS demonstrates that PITX2 and ZFHX3 signals are shared across ancestries [5], the epigenome-wide literature on cardiac tissue is heavily European-weighted, as are the AVNRT and accessory-pathway genetic analyses [56]. Extension of the framework proposed here beyond European-ancestry populations is provisional.

Sixth, the epidemiological estimates vary markedly in between-study heterogeneity. In the most recent meta-analysis, covering 32 studies and more than 34 million participants or analyzed cases, short-term PM_2.5_ exposure standardized to 10 µg/m^3^ was associated with atrial fibrillation at a pooled relative risk of 1.045 (95% CI 1.025–1.066) with negligible heterogeneity (I^2^ = 0.66%), and with sudden cardiac arrest or sudden cardiac death at 1.052 (1.031–1.075; I^2^ = 49.0%); the corresponding long-term PM_2.5_-AF estimate was 1.077 (1.002–1.158) with I^2^ = 99.9%, which the authors themselves regard as limiting interpretation [21]. The short-term associations are thus consistent and modest in magnitude, whereas the long-term estimate is dominated by heterogeneity and should not be used quantitatively.

## 6. Gene–Environment-Epigenetic Interactions: GWAS Loci Meet Environmental Load

### 6.1. The Genetic Landscape of SVTs

Multi-ethnic GWAS have now identified 97 [58], 12 additional [59] and most recently 525 [5] loci meeting genome-wide significance for AF. Across these studies, several recurring biological themes emerge: (1) transcription factors governing atrial development (PITX2, TBX5, PRRX1, ZFHX3); (2) ion channel and gap junction genes (SCN5A, KCNN3, KCND3, CAV1, HCN4); (3) structural proteins (TTN, the giant sarcomeric titin); and (4) genes at the interface of development and electrical patterning (NEURL1, HAND2) [5,58,59].

PITX2, on chromosome 4q25, remains the most significantly associated AF locus across ancestries [5,64]. PITX2 is a paired-like homeodomain transcription factor essential for left-right asymmetry and pulmonary venous myocardium specification during development. In adult atrial tissue, PITX2 represses sinoatrial node gene programs in the left atrium, and its haploinsufficiency creates a unique electrophysiological vulnerability that predisposes to ectopic firing from pulmonary vein sleeves, the most common trigger of paroxysmal AF [65]. ZFHX3 encodes a zinc finger homeobox transcription factor with roles in neural and cardiac differentiation; its AF-associated variants lie in non-coding regions that likely regulate its expression in a tissue-specific manner. The FinnGen study identified over 7600 significant SNPs mapping to 183 loci, with the ZFHX3 region alone harboring 989 significant SNPs, emphasizing the regulatory complexity at this locus [66].

From a cross-ancestry perspective, Yuan et al.’s 2025 cross-population meta-analysis (168,007 AF cases) demonstrated that PITX2 and ZFHX3 variants are shared across populations of different ancestries, while the included proteomics analysis highlighted the TGF-β cellular response pathway as enriched among genes functionally prioritized by integrated GWAS and expression quantitative trait locus (eQTL) analysis [5]. The identification of TGF-β signaling as a proteomic-GWAS convergence point is particularly striking, given TGF-β’s centrality as a mediator of environmentally induced epigenetic fibrotic remodeling.

### 6.2. Gene–Environment Interaction

The conceptual framework of gene–environment–epigenome (G × E × Epi) interaction proposes that genetic variants influence the sensitivity of specific genomic loci to environmental epigenetic remodeling. In the context of AF, several mechanistic scenarios can be envisioned.

First, GWAS loci that map to regulatory regions (promoters, enhancers) are, by definition, sites where transcription factor binding and chromatin accessibility are critical for the regulation of nearby effector genes. Environmental factors that alter DNA methylation or histone modifications at these regulatory regions can therefore amplify or suppress the functional consequences of the associated genetic variants. For instance, a common SNP in the PITX2 locus enhancer might reduce PITX2 transcription under basal conditions to a degree insufficient to cause arrhythmia; however, if the same enhancer becomes hypermethylated in response to PM_2.5_ exposure, the combined reduction in PITX2 expression might cross a pathogenic threshold. This epistatic interaction between inherited regulatory variants and environmentally imposed methylation changes represents a clinically important but experimentally under-explored mechanism.

Second, single-nucleotide variants in DNMT3A, TET2, and other epigenetic modifier genes, originally characterized as somatic clonal hematopoiesis mutations, are now recognized as germline modifiers of epigenetic susceptibility. Individuals carrying functional variants in these epigenetic writer/eraser genes may be more vulnerable to pollution- or lifestyle-induced epigenetic drift at cardiac gene loci, providing a genetic basis for inter-individual variability in the arrhythmic response to environmental stressors [50,54].

Third, GWAS loci in genes directly involved in inflammatory or oxidative stress response (IL6R, identified as a putative causal gene in a 2023 cross-ancestry AF GWAS [67]) create genetic contexts in which environmental pro-inflammatory exposures are particularly damaging. Individuals with variants that amplify IL-6 receptor signaling may mount exaggerated inflammatory epigenetic responses to air pollution, accelerating the fibroinflammatory atrial remodeling that precedes AF.

### 6.3. Towards a Unified G × E × Epi Framework (Figure 1)

Integrating GWAS-identified loci with environmentally responsive epigenetic pathways suggests four convergence points at which inherited and acquired arrhythmic risk may accumulate. The first is the TGF-β/SMAD fibrotic axis, which is under genetic control at multiple loci and is environmentally activated by PM_2.5_, tobacco, obesity and alcohol. The second is the PITX2–pulmonary vein electrophysiological axis, genetically sensitized at 4q25 and potentially further destabilized by promoter methylation under environmental load. The third is the miR-21/SPRY1/ERK fibroblast activation cascade, which intersects with inflammatory environmental stimuli. The fourth is connexin and gap junction remodeling, driven by genetic variants in GJA5 (Cx40) and modulated by methylation under oxidative and inflammatory stress. All four are proposed convergence points. None has been demonstrated to operate as an interaction in human atrial tissue, and none has been examined in AVNRT, AVRT or focal atrial tachycardia.

**Figure 1:**
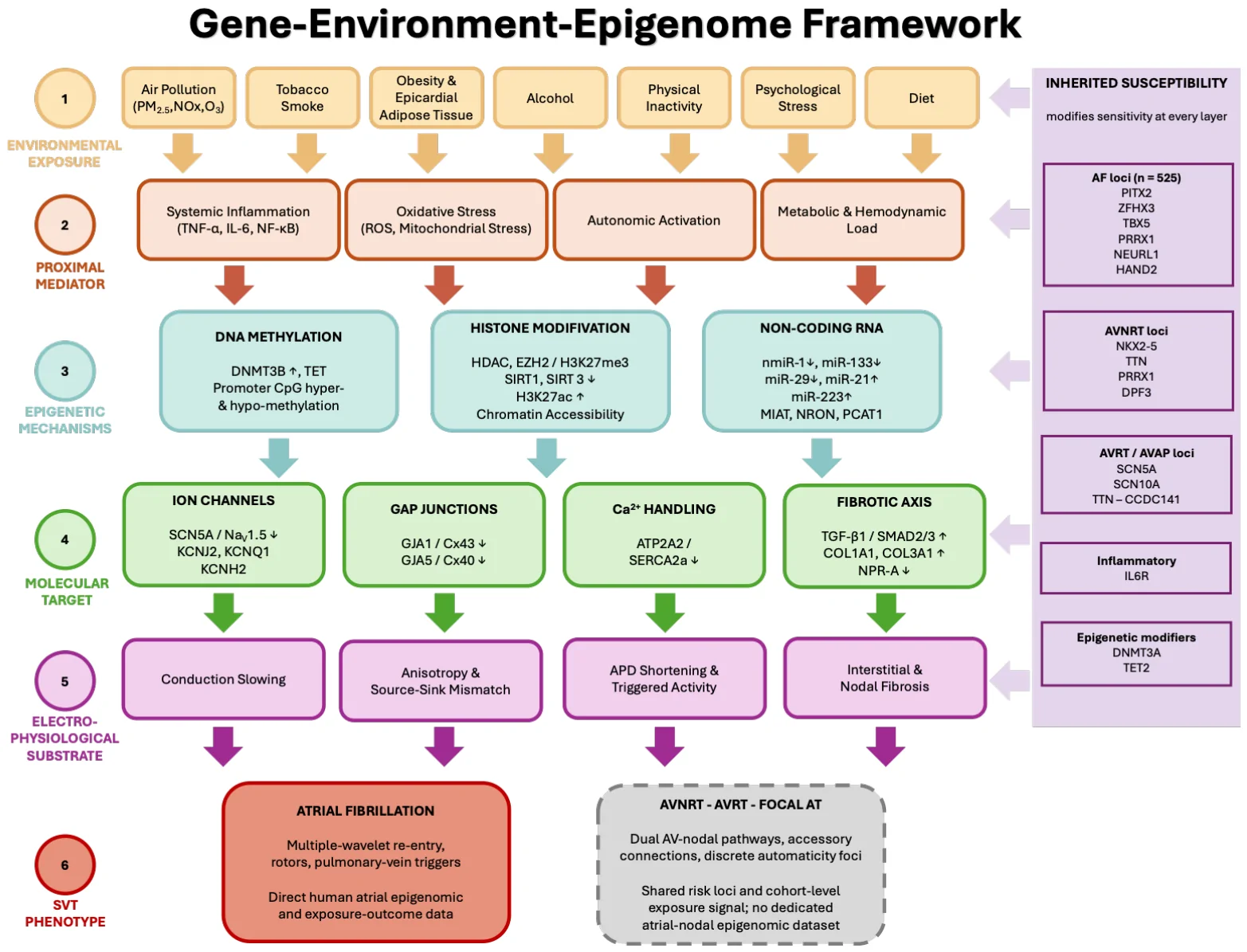
Integrative gene–environment–epigenome (G × E × Epi) framework for supraventricular tachycardia. Six layers connect environmental exposure to arrhythmic phenotype. Layer 1, environmental exposures: ambient air pollutants, tobacco smoke, obesity and epicardial adipose tissue, alcohol, physical inactivity, and psychosocial stress and diet. Layer 2, proximal biological mediators: systemic inflammation, oxidative stress, autonomic activation, and metabolic and hemodynamic load. Layer 3, the epigenetic mechanisms: DNA methylation, histone modification and non-coding RNA networks, with the principal effectors named in each. Layer 4, molecular targets: cardiac ion channels, gap junction proteins, calcium-handling proteins and the TGF-β/SMAD fibrotic axis. Layer 5, the resulting electrophysiological substrate: conduction slowing, anisotropy and source-sink mismatch, action potential duration shortening with triggered activity, and interstitial and nodal fibrosis. Layer 6, arrhythmic phenotype. The right-hand rail shows inherited susceptibility, which modifies sensitivity at every layer, and the G × E × Epi node at which a risk allele and exposure-driven remodeling converge on the same regulatory element. Border and arrow style encode the status of the supporting evidence, not the magnitude of any effect. Solid lines denote relationships supported by direct human data, whether atrial fibrillation-specific or derived from cohort studies of undifferentiated supraventricular tachycardia. Dashed lines denote relationships extrapolated from atrial fibrillation that are mechanistically plausible but empirically unvalidated for AVNRT, AVRT and focal atrial tachycardia. Accordingly, the atrial fibrillation phenotype box is drawn solid and the AVNRT/AVRT/focal atrial tachycardia box dashed. APD, action potential duration; ROS, reactive oxygen species; (downwards arrow), decreased; (upwards arrow), increased.

This framework makes testable predictions: individuals carrying AF-risk alleles at these loci should be disproportionately susceptible to pollution- or lifestyle-induced arrhythmic remodeling, and epigenomic profiling of atrial tissue from such individuals exposed to high versus low environmental loads should reveal differential methylation and chromatin accessibility signatures at the functionally prioritized GWAS loci. Such studies are technically feasible with current multi-omic technology and would yield both mechanistic insights and clinically actionable risk stratification tools.

## 7. Future Directions

### 7.1. Tissue-Specific Epigenomic Profiling in SVT Patients Under Environmental Factors Load

The most pressing research gap in this field is the absence of systematic, tissue-specific epigenomic profiling in SVT patients stratified by environmental exposure burden. Existing human epigenomic studies in AF are constrained by right atrial appendage sampling at cardiac surgery, cell heterogeneity, and the absence of contemporaneous environmental exposure data [2,49].

Future studies should integrate multi-region atrial sampling (right atrial appendage, left atrial wall, pulmonary vein ostia, AV node in relevant cases) with single-cell ATAC-seq and bisulphite sequencing to resolve cell-type-specific chromatin accessibility and methylation landscapes. Such profiling should be paired with detailed environmental exposure histories derived from high-resolution spatiotemporal air quality models, validated biomarkers of smoking exposure (cotinine, AHRR methylation at cg05575921), and metabolic phenotyping. This integrative design would enable identification of environmentally responsive epigenetic signatures in the specific cell populations and anatomical locations most relevant to each SVT subtype.

For AVNRT and AVRT specifically, tissue acquisition during catheter ablation procedures offers an underutilized window into the epigenomics of specialized conduction tissue. Endocardial mapping catheters with integrated tissue sampling capability, or micro-biopsy techniques applicable at the time of ablation, could yield RNA and DNA material sufficient for limited epigenomic profiling. Sample sizes would initially restrict such studies to hypothesis generation, but they would constitute the first direct test of whether the AF framework applies to nodal and accessory-pathway substrate.

### 7.2. Circulating Epigenetic Biomarkers as Exposure and Risk Markers

Circulating cell-free DNA methylation signatures and extracellular vesicle-encapsulated miRNAs represent accessible, minimally invasive epigenomic readouts that could serve dual purposes as exposure markers (reflecting cumulative environmental burden) and risk markers (indicating atrial epigenomic status) [22,50].

The candidate circulating miRNAs listed in Section 4.4 have been validated in AF contexts [44,49], and post-operative AF has been predicted by pre-operative right atrial signatures including reduced miR-133a and miR-29 family members [68]. A multi-marker panel integrating miRNA levels, methylation at pollution-responsive CpG sites (F2RL3, AHRR) and lncRNA signatures could provide a clinically implementable index of risk reflecting both inherited predisposition and cumulative exposure. None has yet been constructed or validated.

The epigenetic clock framework deserves particular mention, and it is instructive precisely because part of the question has already been answered. DNA methylation-agingbiological age diverges from chronological age under conditions of environmental stress, and accelerated epigenetic aging is associated with increased cardiovascular risk [69]; early-life smoking initiation is associated with accelerated methylation-based ageing that partially mediates subsequent cardiovascular risk [70], and smoking leaves reproducible, trans-ethnic methylation signatures [71]. For atrial fibrillation specifically, Roberts et al. examined four epigenetic clocks in 5600 participants from three population-based cohorts with 905 incident AF events over a mean 12.9 years of follow-up. A five-year increment in DNAm GrimAge acceleration was associated with a 19% higher hazard of incident AF (adjusted hazard ratio 1.19, 95% CI 1.09–1.31) and a five-year increment in DNAm PhenoAge acceleration with a 15% higher hazard (1.15, 1.05–1.25), each independent of chronological age and of conventional risk factors. Two-sample Mendelian randomization using genetic instruments for the same measures, however, showed no association with AF [27]. The observational signal is therefore reproducible, and the causal claim is not supported by the available genetic evidence. Epigenetic age acceleration is best regarded at present as an integrative marker of accumulated biological insult that may contribute to risk stratification, rather than as a mechanism to be therapeutically targeted. Whether the same relationship holds for AVNRT, AVRT or focal atrial tachycardia is untested, and given the substantially younger age at presentation of these arrhythmias it should not be assumed.

### 7.3. Epigenetic Therapeutics and Environmental Mitigation

The reversibility of epigenetic modifications is, in principle, the feature that makes them the most pharmacologically attractive targets in cardiovascular medicine. HDAC inhibitors have demonstrated anti-fibrotic effects in preclinical AF models, partly by maintaining histone acetylation at anti-fibrotic gene loci and reducing collagen deposition [49]. DNMT inhibitors and TET-activating compounds could theoretically reverse aberrant DNA methylation at ion channel or gap junction gene promoters, restoring conduction velocity and reducing re-entrant substrates. CRISPR/dCas9-based epigenome editing, targeted delivery of histone-modifying or DNA-methylating enzymatic domains to specific genomic loci, represents a longer-term but mechanistically precise approach [50,54].

Sodium-glucose cotransporter 2 inhibitors (SGLT2i) merit mention as an indirectly epigenetic pharmacological intervention. Beyond their hemodynamic and metabolic effects, SGLT2i appear to modulate miRNA expression patterns relevant to atrial remodeling and reduce epicardial adipose tissue, potentially interrupting the adipose-to-atrial epigenetic inflammatory crosstalk [72].

Critically, any discussion of epigenetic therapeutics must be accompanied by acknowledgement of environmental mitigation as an upstream public health intervention. The epidemiological evidence reviewed here, demonstrating consistent positive associations between PM_2.5_, NO_2_, and tobacco exposure with AF risk at levels below current regulatory thresholds [15,16], provides a strong scientific argument for tighter air quality standards and expanded tobacco control policies as primary arrhythmia prevention strategies. The epigenomic evidence strengthens this argument by providing a mechanistic account of why these exposures matter at the molecular level and why the cardiac epigenome may not fully recover even after exposure reduction.

## 8. Conclusions

In atrial fibrillation, environmental exposures encompassing urban air pollutants (PM_2.5_, NO_2_, ozone), tobacco smoke, obesity, alcohol, psychosocial stress and physical inactivity are associated with modulation of the atrial epigenome through three interlocking mechanisms: DNA methylation changes at ion channel, connexin, and fibrosis-regulatory gene promoters; histone modification-mediated chromatin remodeling that amplifies inflammatory and fibrotic transcriptional programs; and environmentally responsive non-coding RNA networks that post-transcriptionally dysregulate the electrical and structural homeostasis of atrial tissue.

Atrial fibrillation provides the most mechanistically complete model of this process, with GWAS-identified loci clustering in non-coding regulatory regions that are environmentally responsive through epigenetic mechanisms, and with a well-characterized cascade from pollution-induced inflammation to TGF-β/SMAD-mediated fibrosis, connexin remodeling, and sustained re-entry. For AVNRT, AVRT, and focal atrial tachycardias, the epigenomic literature is sparse to the point of near-absence, and intellectual honesty requires that extrapolation from AF to these mechanistically distinct arrhythmias be treated as a working hypothesis requiring dedicated experimental validation rather than an established fact.

The promise of this framework lies in its clinical translational potential. Circulating epigenomic biomarkers, such as miRNA panels, methylation signatures at pollution-responsive CpG sites, epigenetic clock indices, could enable earlier and more precise identification of individuals whose genetic predisposition has been epigenetically amplified by environmental load, permitting targeted preventive intervention. Conversely, epigenetic risk markers could identify individuals for whom personalized environmental counseling (relocation from high-pollution areas, smoking cessation support, structured weight management) would be expected to yield the greatest arrhythmic risk reduction.

## Data Availability

No new data were created or analyzed in this study. Data sharing is not applicable to this article.
